# Evolutionary agroecology: Trends in root architecture during wheat breeding

**DOI:** 10.1111/eva.12749

**Published:** 2018-12-26

**Authors:** Yong‐He Zhu, Jacob Weiner, Ming‐Xi Yu, Feng‐Min Li

**Affiliations:** ^1^ State Key Laboratory of Grassland Agro‐ecosystems, Institute of Arid Agroecology, School of Life Sciences Lanzhou University Lanzhou Gansu Province China; ^2^ Department of Plant and Environmental Sciences University of Copenhagen Frederiksberg Denmark

**Keywords:** crop evolution, evolutionary ecology, group selection, root architecture, seminal root traits, soil resource, wheat cultivars, yield

## Abstract

Root system characteristics determine soil space exploration and resource acquisition, and these characteristics include competitive traits that increase individual fitness but reduce population performance. We hypothesize that crop breeding for increased yield is often a form of “group selection” that reduces such “selfish” traits to increase population yield. To study trends in root architecture resulting from plant breeding and test the hypothesis that increased yields result in part from group selection on root traits, we investigated root growth and branching behavior in a historical sequence of wheat (*Triticum aestivum*) cultivars that have been widely grown in northwestern China. Plants were grown in gel‐filled chambers to examine growth angles, numbers, and lengths of seminal roots, and in soil‐filled chambers under eight soil resource levels for fractal analysis of root system architecture. Yield in field was evaluated at standard and low planting densities. Newer cultivars produced higher yields than older ones only at the higher sowing density, showing that increased yield results from changes in competitive behavior. Seminal root number and growth angles were negatively correlated with yield, while primary seminal root length was positively correlated with yield. Roots of higher‐yielding modern varieties were simpler and less branched, grew deeper but spread less laterally than modern varieties. The fractal dimension of root branching was negatively correlated with the yield of cultivars at all resource levels. Root:shoot ratio was negatively correlated with yield under high soil resource levels. The results are consistent with the hypothesis that the success of wheat breeding for higher yields over past 100 years in northwestern China has been in part due to unconscious group selection on root traits, resulting in smaller, less branched, and deeper roots, suggesting a direction for further increases in crop yield in the future.

## 1 INTRODUCTION

Natural selection increases the fitness of individuals, but this does not always maximize population performance, because “selfish” traits or behaviors, which damage population performance, are often selected (Denison, Kiers, & West, 2003; Weiner, Du, Zhang, Qin, & Li, 2017). Since the primary goal of crop breeding has been to increase population performance (i.e., yield), not to maximize individual fitness, it has been argued that there is potential for increasing crop yields through “group selection” (Denison et al., 2003; Donald, 1981; Weiner, 2003), which is considered by most evolutionary biologists to be absent or rare in nature. We have hypothesized that many of the increases in crop yield to date have been due to inadvertent “group selection” by plant breeders (Weiner et al., 2017). Here, we ask whether changes in root architecture over a century of wheat (*Triticum aestivum* L.) breeding in semi‐arid northwestern China are consistent with this hypothesis.

Most improvements in crops and agricultural practices have focused on shoot biomass, architecture, and grain yield (Gonzalez, Beemster, & Inzé, 2009; Xing & Zhang, 2010). Reduction in the height of cereals has been one of the most successful modifications of shoot traits and one of the most important agricultural innovations of the 20th century, resulting in substantially increased grain production (Khush, 2001; Sasaki et al., 2002). Donald (1968) provided a list of the desirable shoot architectural characteristics for what he called a cereal “ideotype” for intensive production: short stem, few, small, erect leaves, a large, and erect ear. He implied that these traits will benefit population yield in monoculture at the expense of individual performance in a more diverse plant population or community.

Although there is broad agreement that root traits are just as important as shoot traits in ecology and agriculture, plant ecologists and crop breeders have tended to focus on aboveground traits because of the difficulty of observing and measuring/screening belowground traits, but the increased emphasis on plant roots in recent years is changing this. Root architectural traits have important effects on the uptake of water (Uga et al., 2013), nitrogen (Forde, 2014; Kiba & Krapp, 2016) and phosphorus (Lynch, 2011; Péret et al., 2014), and their interactions with neighbors (Cahill et al., 2010). The importance of root systems, specifically root architecture, for crop yield and other agronomic objective is widely appreciated (Den Herder, Van Isterdael, Beeckman, & De Smet, 2010; Dorlodot et al., 2007). Several researchers have suggested that specific root architectural traits can improve soil resource acquisition and benefit crop yield (Comas, Becker, Von Mark, Byrne, & Dierig, 2013; Kong, Zhang, De Smet, & Ding, 2014; Lynch, 1995; Rogers & Benfey, 2015). Special attention has been given to traits contributing to plant productivity under water limitation. These include small fine root diameters, long specific root length, and high root length density (Comas et al., 2013).

Several traits of seminal roots (lateral roots that develop from the radicle and are present in the embryo) of wheat, which largely determine the architecture of the root system at the adult stage, can be conveniently investigated at an early growth stage (Løes & Gahoonia, 2004). These include root growth angle, seminal root number, and length.

While most of the discussion of root traits has focused on abiotic factors limiting plant growth, there is evidence that some root functional traits influencing individual survival and growth in nature may be disadvantageous to a crop population as a whole. A game‐theoretical model predicts that natural selection will result in an overproduction of roots to the detriment of population yield (Zhang, Sun, & Jiang, 1999). Tests of the model have supported the hypothesis that increases in yield have been associated with decreases in root overproduction (Zhu & Zhang, 2013). There is evidence for “overproduction” of roots in competing soybean (Gersani, Brown, O'Brien, Maina, & Abramsky, 2001) and wheat (Y‐H Zhu, unpublished data) plants, and for a negative relationship between root:shoot ratio and wheat yield (Song et al., 2009), which are consistent with the hypothesis (Fang, Liu, Xu, & Li, 2011). A study of nine wheat cultivars developed over the past 100 years in Australia found that root dry matter in the top 40 cm of soil has declined over this period (Siddique, Belford, & Tennant, 1990). This is consistent with the hypothesis that smaller root systems and a lower root:shoot ratio, and therefore less competitive roots, permit more carbon assimilation by shoots in agricultural fields.

Paralleling Donald's (1981) argument for aboveground architectural traits, we hypothesize that changes in root architecture associated with increasing yields over the past century have been due to unconscious group selection through a weakening of “selfish” traits (Dorlodot et al., 2007). Here, we ask whether changes in several root architectural traits over 110 years of wheat breeding in northwestern China are consistent with the hypothesis that cultivar evolution will show increased population yield at the expense of some traits favored by natural selection.

We chose eight cultivars that are or have been widely grown in the region, and which reflect a sequence of increasing yield over 110 years of breeding in the semi‐arid agricultural area of the Loess Plateau in northwestern China. We grew plants in gel‐filled and in soil‐filled containers to examine seminal root traits and root system architecture under different resource levels relevant to local conditions, and ask whether changes over the course of breeding reflect a reduction in competitive rooting traits, that is, whether there is evidence for group selection on root architecture during wheat cultivar evolution. We also evaluate the relationship between root traits on wheat yield in the field at standard and low density and address relationship between individual traits and population yield.

## 2 MATERIALS AND METHODS

### 2.1 Wheat cultivars

Eight wheat cultivars that are or have been widely grown in semi‐arid agricultural areas of the Loess Plateau and represent a sequence of cultivars with increasing yields (Table 1) were selected for this study: Heshangtou (HST), Jinbaoyin (JBY), Gansu96 (GS96), Dingxi24 (DX24), Dingxi35 (DX35), Longchun8139 (LC8139), Longchun8275 (LC8275), and Ganchun25 (GC25). This region has a typical semi‐arid climate within northwestern China, with a 30‐year average precipitation of 168 mm, mean pan evaporation of 938 mm, mean temperature of 14°C, and mean relative humidity of 59% during the wheat growing season.

**Table 1:** The origins and major characteristics of the eight spring wheat cultivars

Cultivar	Time of release	Origin and morphological characteristics
Heshangtou	Before 1900	Long stem, awnless, large numbers of tillers
Jinbaoyin	Before 1900	Long stem, quadrangular spike, short awn
Gansu96	1950s	Long and fine stem, awned
Dingxi24	1963	Long and fine stem, long awn
Dingxi35	1979	Long stem, long awn, similar with DX24
Longchun8139	1986	Long and sturdy stem, awned
Longchun8275	1997	Long stem, awned, similar to LC8139
Ganchun25	2008	Medium‐dwarf stem, awned, compact form

### 2.2 Root traits

While the literature on root traits is extensive, we focus here on a few measurable, genetically fixed architectural traits of wheat roots that are important for plant–plant interactions belowground and that can be evaluated in young plants grown under controlled conditions: seminal root number, length, and angle (Oyanagi, 1994; Sanguineti et al., 2007; Uga, Kitomi, Ishikawa, & Yano, 2015). We also analyze the fractal dimension of the mature root system (Manschadi, Christopher, Hammer, & Devoil, 2010; Wang, Siopongco, Wade, & Yamauchi, 2009) under several resource conditions.

### 2.3 Field experiment

The field experiment was conducted from March to July 2016 at the Experimental Station of Lanzhou University in Yuzhong County, Gansu Province, China (104°09′ E, 35°56′ N, altitude 1,749 m). The field experiment was performed on homogeneous farmland soil using a split‐plot randomized complete block design with two planting densities in main plots: 256 seeds/m^2^ (the standard seeding rate for this region) and 128 seeds/m^2^ (half the standard planting density), and eight cultivars in subplots. Each plot measured 1.5 m × 1.5 m, and the spacing between neighboring plots was 0.5 m. Following a basal dose of nitrogen (120 kg/ha), phosphorus (60 kg/ha), and potassium (48 kg/ha), wheat grains were sown at a depth of 4 cm in a uniform grid pattern by hand through a 1.5 m × 1.5 m frame with a grid of nylon wires forming the 24 × 24 grid pattern, giving 256 grains/m^2^, and in alternate rows to obtain 128 grains/m^2^. Each treatment was replicated three times. At 121 days after sowing, we harvested a centrally placed 1‐m^2^ subplot within each plot to determinate grain yield.

### 2.4 Gel‐filled chamber experiment

The numbers and growth angles of seminal roots of wheat seedlings were measured using gel‐filled root observation chambers as described by Bengough et al. (2004). Chambers were constructed from two plates, each measuring 400 × 300 × 3 mm. Sterilized agar was poured into the 400 × 300 × 8 mm chambers. Grains of each cultivar were graded, removing the largest and smallest, to select a uniformly sized sample that was near the median size for each cultivar.

Seeds were surface‐sterilized using 75% alcohol and put into sterile deionized water for a few hours. They were then placed on wet blotting paper and kept at 25°C for 1 day to promote germination. Two germinated grains were placed on the top edge of the gel in a vertical chamber with 10‐cm spacing. The grains were oriented vertically with the radicle facing downwards. The gel‐filled chambers were arranged in a complete randomized block with three chambers per cultivar in a light incubator at 20°C and 75% relative humidity in the dark until the first leaf emerged. They were then cultured under a light intensity of approximately 700 lx at the leaf surface at 20°C under 12/12‐hr dark/light conditions. The chambers were covered with silver paper, except during scanning.

**Figure 1:**
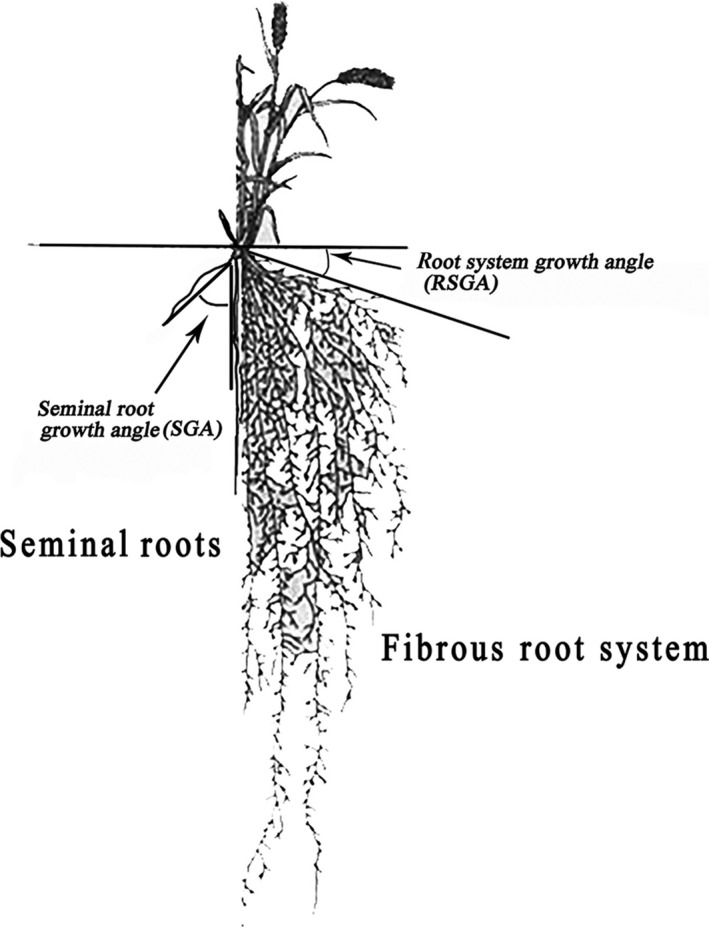
Drawing of wheat roots, modified to show growth angles in wheat seminal roots (left), and root system growth angles in the fibrous root system (right)

The roots were scanned using a root scanner (Epson Expression 10000XL; Epson, Long Beach, CA, USA) every 2 days after the first leaf emerged for 8 days (five times). The growth angles (Figure 1) of individual root axes belonging to the first and last pairs of seminal roots were mapped. The growth angles were calculated and recorded, as were the total numbers of seminal roots and seminal root lengths using ImageJ (National Institutes of Health, NIH, Bethesda, CA, USA) on the 8th day.

### 2.5 Soil‐filled chamber experiment

The soil‐filled chamber experiment was conducted from April to July 2014. Eight soil‐filled chambers (40‐cm wide × 40‐cm deep × 4‐cm thick), constructed using nylon net (200 mesh, 74 μm), were placed in a box (60‐cm long × 40‐cm wide × 42‐cm deep), and the chambers were supported in a square and upright position by wooden boards (4‐cm thick) after being filled with a soil mixture consisting of sifted nutrient‐poor loess and vermiculite (75%:25%). The soil was saturated (field water capacity [FC] = 28.8%) with nutrient solution 3 days before planting. Two grains of each cultivar were planted in the middle of each root chamber at a depth of 3 cm, and one plant was retained after seedling emergence.

A split‐plot experimental design was used, with the main blocks being two water conditions: high water (80% FC) and low water (40% FC). The 80% FC treatment was a watering cycle from 90% FC to 70% FC, and 40% FC was a cycle from 50% FC to 30% FC. Whole plots were the eight wheat cultivars, and the subplots were four nutrient conditions: +N (nitrogen) + P (phosphorus; 0.2 g N and 0.05 g P/kg·dry soil), −N+P (0.05 g P/kg·dry soil), +N−P (0.2 g N/kg·dry soil), −N−P (No N or P, Control). NH_4_NO_3_ and KH_2_PO_4_ were used to supply the nutrients, and K_2_SO_4_ was used to balance the potassium under P‐deficient conditions. Each treatment had seven replicates.

After 117 days, plant shoots were harvested and separated into leaves, stems (including leaf sheaths), and mature spikes, and all plant material was dried for 48 hr at 80°C and then weighed. Soil blocks with roots were removed from the chambers carefully and then placed flat on the ground. A black‐painted pin board with the same dimensions as the chamber was positioned on the open chamber so that the pins penetrated the soil block. The spatial orientation of the root system was maintained by inversion of the intact root system on the pin board. The pins (2 mm diameter × 50 mm length) within the pin board were arranged in a grid pattern with pins spaced 18 mm apart. After carefully washing the soil mixture from the roots, digital photographs of each whole root system were taken with a digital camera mounted on a tripod. The images were converted to high‐contrast black‐and‐white pictures using Photoshop CC software (Adobe Systems Incorporated, San Jose, CA, USA). Following the digital imaging, roots were removed from the pin board, oven‐dried, and weighed. The root system architecture was quantified using fractal analysis under different soil resource conditions.

### 2.6 Fractal analysis of root architecture

Fitter (1987) proposed a topological approach for the analysis of root branching patterns, based on the numbers and spatial arrangements of root links. Fractal geometry is a quantitative method of describing many complex natural objects (Falconer, 2004), which can summarize important aspects of such patterns. Root systems are fractal objects because the repetitive branching of the roots leads to a high degree of self‐similarity, which is the fundamental characteristic of fractal geometry. Fractal dimension is an index of the “space filling” properties of the root system and encompasses both topological and geometric root characteristics. A higher fractal dimension reflects a more highly branched root system. Fractal dimension of root architecture has significant variation among genotypes in several crop species (Manschadi et al., 2010; Wang et al., 2009).

Fractal dimensions were calculated from the root system images using the box‐counting method (Tatsumi, Yamauchi, & Kono, 1989). In this procedure, a grid is superimposed on the root system image and the numbers of squares intercepted by roots at various grid square sizes are counted. The fractal dimension is then estimated by fitting the following linear regression model:

logN(r)=−Dlogr+logK,

where *N*(*r*) represents the number of squares intercepted by roots, *r* represents the width of the square, and *K* is a constant. The slope of the regression line is an estimate of the fractal dimension. By definition, if a two‐dimensional object is fractal, the value of fractal dimension must be >1 and ≤2. The length of the square side used varied from 2 to 10 mm in seven steps. Fractal dimensions were estimated for the entire root system and for individual root system sections formed by dividing the root system images into nine (120 × 120 mm) segments. A computer program was written in MATLAB (MathWorks, Natick, USA) to automate the fractal dimension calculations (see Data accessibility).

### 2.7 Statistical analyses

Yield in field experiment was used to reflect and quantify the process of wheat cultivar evolution when examining the trends in root architectural traits during breeding. Data were analyzed using generalized linear mixed models (Stroup, 2012). Mean comparisons were made with Tukey's test at *p* = 0.05 significance level. Visual inspection of residuals showed that the relationships between yield in the field at the standard density and root variables were consistent with the assumptions of the statistical models. All statistical analyses were performed using GenStat for Windows (version 17; VSN International, Hemel Hempstead, UK).

## 3 RESULTS

### 3.1 Changes in field yield

**Figure 2:**
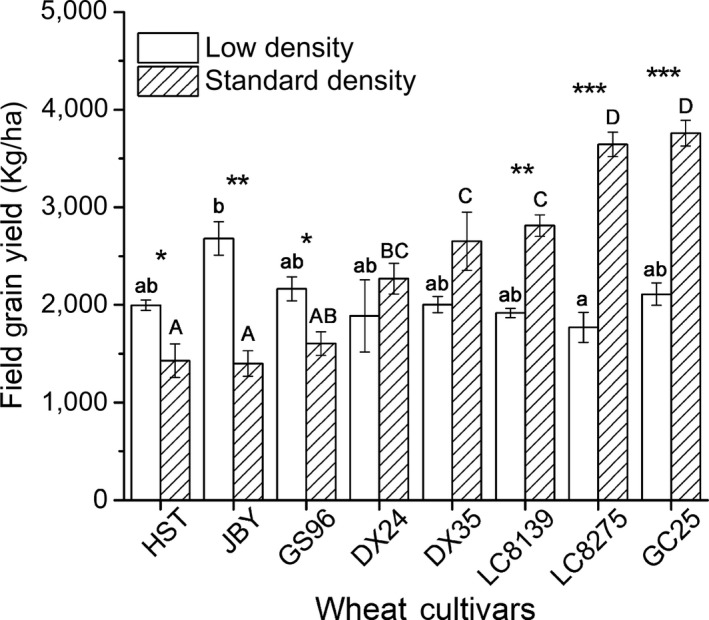
Field yield of the eight cultivars at low (128 grains/m^2^, open columns) and standard density (256 grains/m^2^, filled columns), ranked by the latter. Bars indicate ± standard errors. Different uppercase letters denote significant differences at the standard density; lowercase letters refer to differences at the low density (*p* = 0.05). For differences between the lower and standard density of each cultivar: *p ≤ 0.05, **p ≤ 0.01, ***p ≤ 0.001

The field yield increased continually and significantly with cultivar release date at the standard planting density for this region, but only two cultivars (JBY and LC8275) had yields significantly different from each other at the low sowing density (Figure 2). The newest cultivars, which produced the highest yields at the standard density, produced low yields at the lower density, while the oldest cultivars produced significantly higher yields at low than at high density.

### 3.2 Correlations between seminal root traits and yield in the field

**Figure 3:**
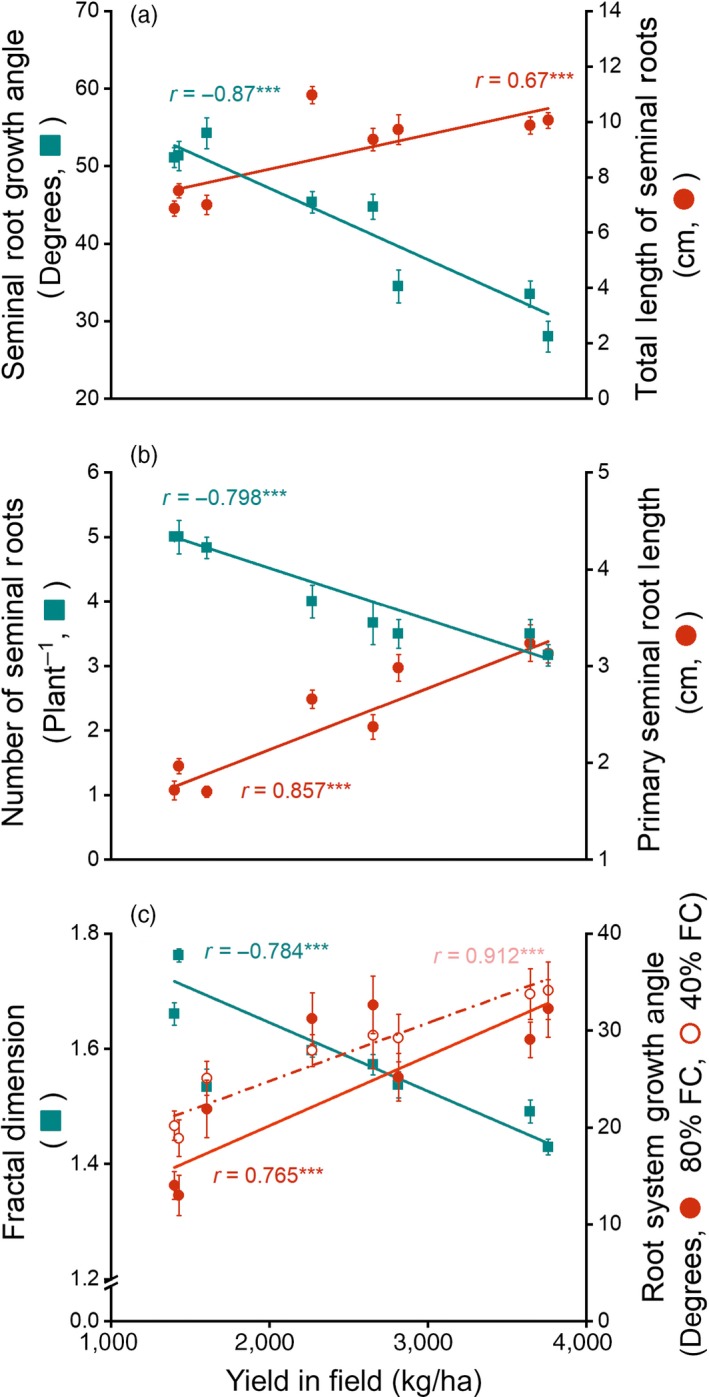
Seminal roots traits versus yield for eight cultivars of spring wheat spanning over 100 years of breeding. (a) Seminal root growth angle and total length of wheat seminal roots in gel‐filled chambers; (b) numbers of seminal roots and primary seminal root lengths in gel‐filled chambers; (c) fractal dimension and root system growth angle in soil‐filled chambers; FC: field capacity. Data are presented as means ± standard errors for visual clarity, but lines, correlation coefficients (*r*), and *p* values are based on the data themselves (see Data accessibility), ***p ≤ 0.001

Field yield was significantly and positively correlated with total and primary seminal root length (*r* = 0.67, *p* < 0.001; *r* = 0.857, *p* < 0.001, respectively). The number of seminal roots and the seminal root growth angles, the angle between primary seminal root and the last seminal root, increased during cultivar evolution and had significantly negative correlations with field yield (number: *r* = −0.798, *p* < 0.001; angle: *r* = −0.87, *p* < 0.001, respectively; Figure 3).

Correlations among seminal root traits (Table 2a) showed that the seminal root growth angles were significantly negative correlated with the total seminal root length (*r* = −0.68, *p* < 0.001) and the primary seminal root length (*r* = −0.873, *p* < 0.001), but was positively correlated with the number of seminal roots (*r* = 0.732, *p* < 0.001). Total seminal root length was negatively correlated with the number of seminal roots (*r* = −0.819, *p* < 0.001) and positively correlated with primary seminal root length (*r* = 0.87, *p* < 0.001). The number of seminal roots was significantly negatively correlated with the primary seminal root length (*r* = −0.872, *p* < 0.001) over the course of cultivar evolution.

**Table 2:**
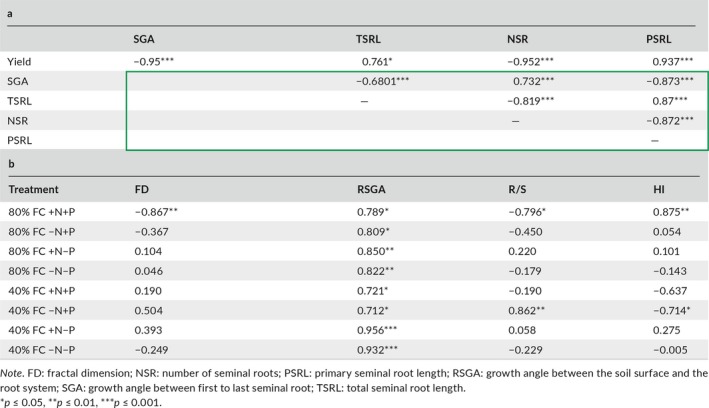
Correlations among seminal root traits, root architectural traits, root: shoot ratio (R/S), and harvest index (HI). (a) Correlations between means of seminal root traits and field yield, and among seminal root traits in gel‐filled chambers (Green box). (b) Correlations between field yield and root system architectural traits, R/S and HI under conditions of 80% FC and 40% FC in soil‐filled chambers with added nitrogen and phosphorus (+N+P), nitrogen only (+N−P), phosphorus only (−N+P), or no fertilization (−N−P)

### 3.3 Resource levels and root architecture traits

The growth angle of root system (Figure 1), the angle between surface of the soil and root system, was positively correlated with field yield at both water levels (low water availability: *r* = 0.912, *p* < 0.001; high water availability: *r* = 0.765, *p* < 0.001).

**Figure 4:**
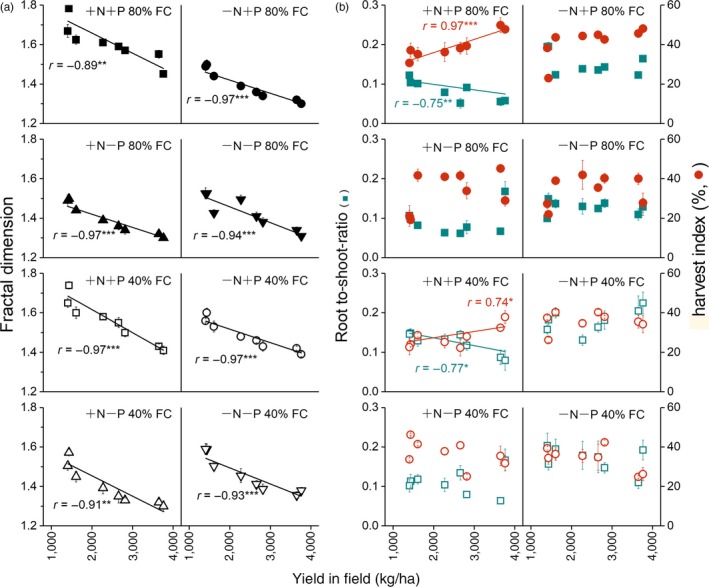
Fractal dimension and dry‐matter allocation of cultivars of spring wheat under different soil resource treatment in soil‐filled chambers versus field yield for eight cultivars of spring wheat developed over 110 years of breeding. (a) Fractal dimensions of whole root systems, (b) root: shoot ratio (R/S), and harvest index (HI) of wheat cultivars grown in soil‐filled chambers under conditions of 80% and 40% field capacity (FC), with added nitrogen and phosphorus (+N+P), nitrogen only (+N−P), phosphorus only (−N+P), or no fertilization (−N−P) at maturity. Error bars are ±standard errors. *p ≤ 0.05, **p ≤ 0.01, ***p ≤ 0.001

The fractal dimension was affected by soil resource conditions and decreased significantly with field yield, which increased during cultivar evolution (Figure 4a). There was no significant effect of N (*p* = 0.16) or an N × water interaction (*p* = 0.105) on fractal dimension, but other factors and interactions did have significant effects on fractal dimension (*p* < 0.05; Supporting Information Table S1). The negative correlations with field yield were not changed by soil resource treatments. The maximal fractal dimension of each cultivar occurred at high water and nutrient levels.

Dry‐matter distribution results showed that the root‐to‐shoot ratio (R/S, Figure 4b) decreased significantly (*p* < 0.05) and the harvest index (HI, Figure 4b) increased significantly (*p* < 0.05) over the course of wheat breeding, but only under high soil resource levels.

### 3.4 Root traits and field yield

Correlation analyses of the data for the field experiment, gel‐filled chamber experiment, and soil‐filled chamber experiment under eight soil resource conditions (Table 2) showed that yield was negatively correlated with seminal root growth angle (*r* = −0.95, *p* < 0.001) and with the number of seminal roots (*r* = −0.952, *p* < 0.001) and positively correlated with total seminal root length (*r* = 0.761, *p* < 0.05) and primary seminal root length (*r* = 0.937, *p* < 0.001). Fractal dimension and root:shoot ratio at 80% FC +N+P conditions were negatively correlated with field yield (*r* = −0.867, *p* < 0.01; *r* = −0.796, *p* < 0.05, respectively), while harvest index was positively correlated with field yield (*r* = 0.875, *p* < 0.01). The root system growth angle was positively correlated with field yield in all treatments.

**Figure 5:**
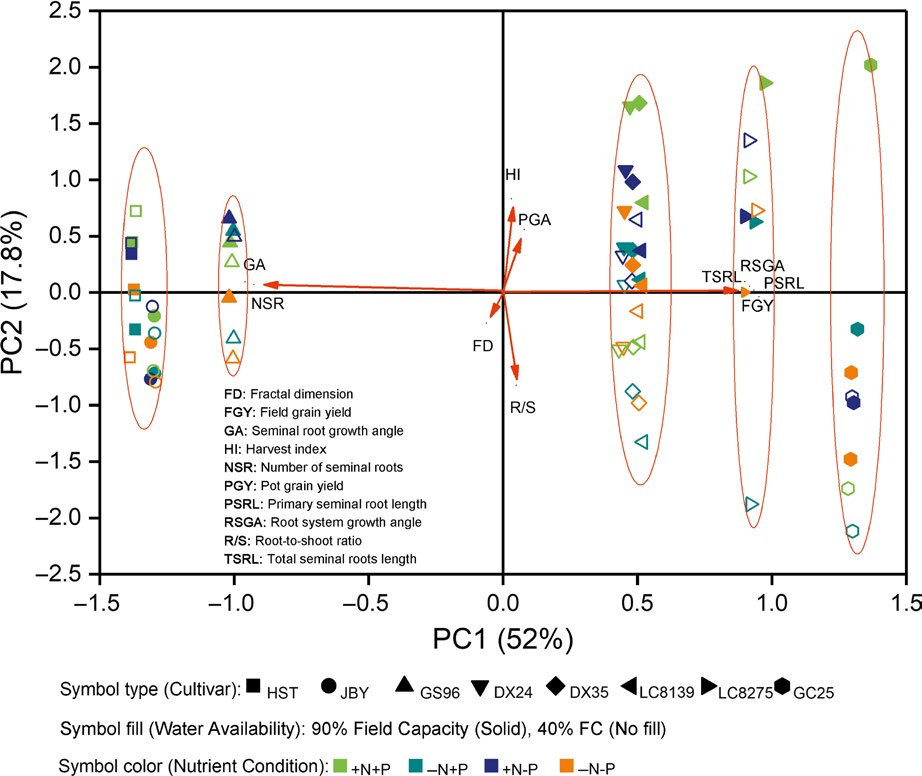
Results of principal component analysis based on root architectural traits and biomass characteristics of eight cultivars of spring wheat

In a principal component analysis (PCA; Figure 5) based on root architectural traits and biomass, PC1 reflects root characters that are highly correlated with field yield and seminal root length and negatively correlated with seminal root number and growth angle. PC2 reflects allocation traits, indicating interactions between dry‐matter distribution and soil resource treatment. PC2 is positively correlated with HI and pot grain weight and negatively correlated with root:shoot ratio. The eight cultivars were clearly separated into five groups on PC1, showing differences in root traits and field yield among the five groups. Fractal dimension, root‐to‐shoot ratio, harvest index, and pot yield are correlated with PC2, showing the influence of resource conditions, and that the low‐yielding varieties were more stable than high‐yielding varieties (Figure 5, Supporting Information Figure S1).

## 4 DISCUSSION

Increasing yield has been the primary goal of cereal breeding, and this depends in part on the interaction between individual and population behavior. This study focuses on changes in individual root traits by investigating eight cultivars spanning a period from before 1900 until 2008, in an attempt to test a hypothesis on how changes in root architecture may have contributed to increases in yield.

Yield of wheat cultivars grown at the standard sowing density has increased continually and significantly over the course of breeding, but no such increase in yield is observed when the crop is grown at a much lower density (Figure 2). This is consistent with the hypothesis that high yield in modern crop varieties results from their ability to be productive at high densities under high‐resource conditions (Tokatlidis & Koutroubas, 2004). In this study, we used the field yield of cultivars grown at the standard sowing density to quantify the effects of wheat cultivar development on several root traits.

### 4.1 More optimized root traits are consistent with “group selection”

Our results show strong and significant correlations between several root traits and field yield during wheat cultivar evolution. The seminal root traits we measured are genetically determined with very limited plasticity (Dorlodot et al., 2007; Sanguineti et al., 2007) and therefore clearly reflect changes due to artificial selection. According to our hypothesis, root traits have been selected to optimize architecture at the population, not individual, level to increase yield. Seminal growth angles narrowed, the number of seminal roots declined, and primary seminal root length increased during the development of higher‐yielding cultivars. Primary seminal root length reflected total seminal root length (*r* = 0.87, *p* < 0.001) such that total seminal root length increased with increasing yield.

The changes in seminal root traits are consistent with reduced individual competitiveness over the course of wheat evolution in this region (Song et al., 2010). Fewer, longer seminal roots with narrower growth angles resulted in the formation of a narrower, deeper, and simpler root system, which overlaps less with neighbors, reducing competition among neighboring individuals (De Parseval, Barot, Gignoux, Lata, & Raynaud, 2017). These trends are consistent with our hypothesis of weakening “selfish” root traits (Weiner et al., 2017).

Although breeders have selected for higher yield empirically, without focusing on underground characteristics, the higher‐yielding cultivars had narrower and therefore less competitive root systems with lower fractal dimensions, even though many breeders argued that a larger root system would be advantageous for capturing nutrients. These changes are consistent with the hypothesis that breeders were inadvertently practising “group selection” during the breeding process. In recent years, breeders have begun to use group selection proactively at the later, yield‐testing stages of breeding (Murphy, Swanton, Van Acker, & Dudley, 2017). The trade‐offs among the seminal root growth angles, the number of seminal roots and the primary seminal root length (Figures 3 and 5; Table 2) observed during cultivar evolution, suggest that there has been a trade‐off between root traits favored by group selection and those favored by natural selection. Group selection implies that the growth and development of individuals have fewer negative effects on neighboring individuals in the crop population. Narrower and deeper roots benefit population performance at the expense of individual fitness.

### 4.2 Root traits and resource uptake

In general, the wheat cultivars that have wide seminal root growth angles and large numbers of seminal roots form large, wide root systems, placing most of the roots close to the soil surface (Supporting Information Table S2). Large shallow rooting systems are advantageous for individuals in taking up nutrients from fertilizers and water from rainfall, but overlap among such large shallow roots will also results in an “overproduction” of roots at the population level, which has been called a “tragedy of commons” (Gersani et al., 2001; Zhang et al., 1999), resulting in lower population yield.

The weakened competitive traits could reduce the capture of resources by individual plant in competition, but if all individuals are less competitive, the stand will benefit and produce higher yield if the crop density and resource levels are high enough. The results show that the relationships between yield, root:shoot ratio, and harvest index were influenced by soil resource levels (Tester & Langridge, 2010). Root:shoot ratio and harvest index were significantly related to increases in yield only under high N and P levels (Figure 4b). This is consistent with previous research showing that the basis for higher yields is in large part a decrease in root:shoot ratio and an increase in harvest index (Fang et al., 2011; Siddique et al., 1990; Song et al., 2009, 2010).

Water is crucial to all physiological processes, and large quantities are required to produce high yields. The results of the soil chamber experiment under different soil resource conditions demonstrate that root system architecture was altered by soil water conditions. We hypothesize that root architectural differences and trends among the eight cultivars may result in different contrasting adaptations to cope with water limitations. The genotypic variation in root architecture documented here may have significant functional implications for the timing and amount of soil water uptake. The low‐yielding cultivars form a large and shallow root system with greater potential for water extraction from the top soil layers, but this architecture results in strong competition among neighboring plants. This drought‐adaptive strategy may optimize the timing of soil water extraction for growth and survival in nature, while reducing the total amount of water taken up and used to produce yield later in development (Comas et al., 2013; Zaman‐Allah, Jenkinson, & Vadez, 2011). The compact, narrow, and deep‐rooted architecture of the higher‐yielding varieties appears to reduce water use early in the season and increase access to water from the deeper soil layers later, during the reproductive phase (Condon, Richards, Rebetzke, & Farquhar, 2004; Zaman‐Allah et al., 2011). Farmers in most dry regions of the world cannot provide additional water for their crops. Therefore, a simple, efficient, and more vertical root architecture has important effects on plant water and nutrient uptake under drought conditions. This is also consistent with our group selection hypothesis. “Saving” water in a dry environment would not be a good strategy for individuals in nature, as saved water will be taken up by other individuals, but it can be a good strategy for agricultural purposes, where the farmer controls the agricultural plant community.

### 4.3 Relationships between root traits and population yield

Root traits are fundamental for the production of yield because they determine water and mineral nutrient uptake, which are essential for growth and yield formation (Comas et al., 2013; Manschadi, Christopher, & Hammer, 2006). Newer cultivars had root traits suited to “group interest” and produce high grain yield under fertile conditions, but older cultivars had more optimal root architectural characteristics at the individual level, and are more adapted to surviving in extremely resource‐limited condition (Ehdaie, Layne, & Waines, 2012; Kong et al., 2014). A crop population composed of “altruistic” individuals will produce greater yields under high‐resource levels, but may be more sensitive to low soil resource levels (Lipiec, Doussan, Nosalewicz, & Kondracka, 2013), increasing the risk of crop failure under unsatisfactory resource conditions.

Root architecture determines a plant's ability to intercept and absorb water and mineral nutrients and is depended on root carbon input. Increased carbon allocation to roots requires reduced allocation to photosynthetic shoot tissues and reproductive organs. The PCA results point toward the traits of cultivars that produced high yield (Figure 5). The history of wheat evolution is reflected in PC1, showing the increase in yield increasing during cultivar evolution and changes among root functional traits of the eight wheat cultivars. PC2 indicates differences in productivity under different soil resource conditions.

The results are consistent with our main hypothesis that the increases in wheat yields in northwestern China have been due in part to a reduction in “selfish” root traits, which increase individual fitness in competition but are detrimental to population production (Tester & Langridge, 2010).

### 4.4 Alternative hypotheses

While the results are consistent with our hypothesis, correlation does not necessarily imply a cause and effect relationship, and it is possible that the traits we have examined are genetically, physiologically, or phylogenetically linked with other traits we did not investigate, and which could be the causes of the observed increases in yield. For example, (a) the increases in yield may be primarily due to aboveground traits, not the root traits we have studied here, although there is evidence that above‐ and belowground traits are not highly linked and can evolve independently (Weiner et al., 2017). (b) While several of the traits we looked at are genetically fixed, others are not, and the latter group could behave differently when plants are grown in competition, rather than in isolation, as measuring them requires. (c) Simpler, less branched and more vertical roots could be result of adaptation to obtaining water from deeper soil layers (Kembel & Cahill, 2011), rather than reducing competition among roots, but in this case we would expect the modern cultivars to produce higher yields than the old cultivars at low as well as standard density. (d) Increased fertilizer levels allow for cultivars with less developed root systems. This could account for smaller root systems, but not the reduction in fractal dimension, the deeper, more vertical seminal roots, or the poor performance at low density. Fertilizers are added from above, not below the rooting zone, and there is evidence that deeper roots are beneficial for nitrogen uptake at low, but not high, nitrogen levels (Comas et al., 2013). Additional studies are needed to test these alternatives against our hypothesis.

## 5 CONCLUSIONS

Over the course wheat breeding in northwestern China, several root functional traits have been modified significantly in ways that are consistent with group selection on the traits investigated. As has been documented for shoot traits, the weakening of competitive traits has allowed for higher planting densities, which produce high yields. The trade‐offs among seminal root traits appear to have benefitted population production at the expense of individual fitness. Fewer, longer seminal roots with narrower growth angles have resulted in narrower, deeper root systems with simpler architecture and lower fractal dimension, improving the efficiency of dry‐matter allocation under agronomic conditions.

High yields are dependent on high‐resource levels, but traits that produce high yields of cultivars under optimal conditions may limit tolerance of adverse environmental conditions, increasing the risk of crop failure if conditions in the field are very far from optimal.

## CONFLICT OF INTEREST

None declared.

## AUTHORS’ CONTRIBUTIONS

Y.‐H.Z. and F.‐M.L. conceived the ideas and designed the methodology; Y.‐H.Z. and M.‐X.Y. collected the data; Y.‐H.Z. and F.‐M.L. analyzed the data; and Y.‐H.Z. and J.W. led the writing of the manuscript. All authors contributed to the drafts and gave final approval for publication.

## Data Availability

The code for fractal dimension calculation is stored in the figshare: https://figshare.com/s/aaddd374cd9e1ca8b431. Data from this study are available from the figshare: https://doi.org/10.6084/m9.figshare.5813475.v4
