# Supplementary material for: Evolutionary agroecology: Trends in root architecture during wheat breeding
Source: Evol Appl. 2018 Dec 26;12(4):733–43. doi: 10.1111/eva.12749 (PMC6439874; doi:10.1111/eva.12749)
Supplement: Supplementary file 1 [file EVA-12-733-s001.docx]

***Supplementary Information***

Table S1 The effects of treatments and cultivars on biomass, root to shoot ratio (R/S), harvest index (HI) and fractal dimension (FD). Analyses of Generalized Linear Mixed Models were used in analyses with treatments and cultivars as fixed factor.

|  |  | Underground  Biomass | | Aboveground Biomass | | Grain Weight | | R/S | | HI | | FD | |
| --- | --- | --- | --- | --- | --- | --- | --- | --- | --- | --- | --- | --- | --- |
| Fixed term | n.d.f. | F statistic | F pr | F statistic | F pr | F statistic | F pr | F statistic | F pr | F statistic | F pr | F statistic | F pr |
| Cultivar (C) | 7 | 21.04 | <0.001 | 35.33 | <0.001 | 47.87 | <0.001 | 6.89 | <0.001 | 20.24 | <0.001 | 59.89 | <0.001 |
| Water (W) | 1 | 33.1 | <0.001 | 26.97 | <0.001 | 200.71 | <0.001 | 93.4 | <0.001 | 56.24 | <0.001 | 33.19 | <0.001 |
| Nitrogen (N) | 1 | 36.01 | <0.001 | 532.51 | <0.001 | 572.32 | <0.001 | 181.51 | <0.001 | 4.85 | 0.028 | 1.98 | 0.16 |
| Phosphorus (P) | 1 | 1119.68 | <0.001 | 1553 | <0.001 | 1924.74 | <0.001 | 21.65 | <0.001 | 3.69 | 0.055 | 503.35 | <0.001 |
| C*W | 7 | 11.19 | <0.001 | 3.07 | 0.004 | 14.29 | <0.001 | 4.7 | <0.001 | 16.17 | <0.001 | 22.26 | <0.001 |
| C*N | 7 | 5.81 | <0.001 | 14.94 | <0.001 | 22.51 | <0.001 | 3.98 | <0.001 | 8.72 | <0.001 | 5.65 | <0.001 |
| W*N | 1 | 2.29 | 0.131 | 4.61 | 0.032 | 64.17 | <0.001 | 14.26 | <0.001 | 0.05 | 0.824 | 2.64 | 0.105 |
| C*P | 7 | 9.73 | <0.001 | 14.34 | <0.001 | 13.64 | <0.001 | 2.37 | 0.022 | 5.39 | <0.001 | 9.69 | <0.001 |
| W*P | 1 | 24.8 | <0.001 | 7.18 | 0.008 | 136.72 | <0.001 | 5.6 | 0.018 | 81.6 | <0.001 | 7.79 | 0.006 |
| N*P | 1 | 129.62 | <0.001 | 591.76 | <0.001 | 631.32 | <0.001 | 8.69 | 0.003 | 3.17 | 0.076 | 50.56 | <0.001 |
| C*W*N | 7 | 9.69 | <0.001 | 1.77 | 0.092 | 5.32 | <0.001 | 3.72 | <0.001 | 2.61 | 0.012 | 9.05 | <0.001 |
| C*W*P | 7 | 11.19 | <0.001 | 3.42 | 0.001 | 19.87 | <0.001 | 4.44 | <0.001 | 13.09 | <0.001 | 28.39 | <0.001 |
| C*N*P | 7 | 6.05 | <0.001 | 10.77 | <0.001 | 14.31 | <0.001 | 6.49 | <0.001 | 4.6 | <0.001 | 20.94 | <0.001 |
| W*N*P | 1 | 0.81 | 0.368 | 7.55 | 0.006 | 89.64 | <0.001 | 0.14 | 0.712 | 19.53 | <0.001 | 17.95 | <0.001 |
| C*W*N*P | 7 | 7.8 | <0.001 | 3.39 | 0.002 | 6.38 | <0.001 | 5.66 | <0.001 | 2.69 | 0.01 | 12.65 | <0.001 |

Table S2 Fractal dimensions (FDs) for the individual 120×120 mm sections of the wheat root systems grown in soil-filled root chambers under conditions of 80% water capacity with added nitrogen and phosphorus (+N+P). T, M, and B indicate top, middle, and bottom layers, respectively; L, C and R represent left, centre and right segments, respectively, within each layer.

| Cultivar | FD of section | | | | | | | | | |
| --- | --- | --- | --- | --- | --- | --- | --- | --- | --- | --- |
|  | TL | | TC | TR | ML | MC | MR | BL | BC | BR |
| HST | | 1.76 | 1.79 | 1.75 | 1.78 | 1.82 | 1.78 | 1.80 | 1.79 | 1.81 |
| JBY | | 1.68 | 1.69 | 1.68 | 1.70 | 1.72 | 1.73 | 1.69 | 1.74 | 1.73 |
| GS96 | | 1.61 | 1.65 | 1.62 | 1.61 | 1.67 | 1.62 | 1.58 | 1.65 | 1.62 |
| DX24 | | 1.59 | 1.63 | 1.62 | 1.62 | 1.65 | 1.62 | 1.60 | 1.61 | 1.60 |
| DX35 | | 1.46 | 1.60 | 1.44 | 1.56 | 1.61 | 1.58 | 1.40 | 1.60 | 1.53 |
| LC8139 | | 1.42 | 1.58 | 1.41 | 1.47 | 1.60 | 1.55 | 1.50 | 1.59 | 1.53 |
| LC8275 | | 1.42 | 1.57 | 1.38 | 1.43 | 1.62 | 1.35 | 1.42 | 1.62 | 1.35 |
| GC25 | | 1.30 | 1.50 | 1.32 | 1.41 | 1.62 | 1.48 | 1.45 | 1.60 | 1.46 |
| LSD (p=0.05) | | 0.127 | 0.084 | 0.113 | 0.152 | 0.101 | 0.163 | 0.121 | 0.144 | 0.116 |

TABLE S3 The eigenvalues and eigenvectors of PCA. (A) The explained total variance; (B) Component Matrix. GA: Seminal root growth angle; NSR: Number of seminal roots; TSRL: Total seminal roots length; PSRL: primary seminal root length; HI: Harvest index; R/S: Root to shoot ratio

FD: Fractal dimension; RSGA: Root system growth angle.

| A Total Variance Explained | | | | | | |
| --- | --- | --- | --- | --- | --- | --- |
| Component | Initial Eigenvalues | | | Extraction Sums of Squared Loadings | | |
|  | Total | % of Variance | Cumulative % | Total | % of Variance | Cumulative % |
| 1 | 5.200 | 51.997 | 51.997 | 5.200 | 51.997 | 51.997 |
| 2 | 1.785 | 17.848 | 69.844 | 1.785 | 17.848 | 69.844 |
| 3 | 1.378 | 13.775 | 83.620 |  |  |  |
| 4 | .560 | 5.601 | 89.221 |  |  |  |
| 5 | .504 | 5.039 | 94.259 |  |  |  |
| 6 | .317 | 3.173 | 97.432 |  |  |  |
| 7 | .184 | 1.843 | 99.275 |  |  |  |
| 8 | .054 | .543 | 99.818 |  |  |  |
| 9 | .014 | .141 | 99.959 |  |  |  |
| 10 | .004 | .041 | 100.000 |  |  |  |
| Extraction Method: Principal Component Analysis. | | | | | | |

B Component Matrix

|  | Component | |  |
| --- | --- | --- | --- |
|  | 1 | 2 | |
| FD | -0.062 | -0.277 | |
| R/S | 0.049 | -0.831 | |
| HI | 0.031 | 0.827 | |
| Field Yield | 0.951 | -0.038 | |
| GA | -0.959 | 0.09 | |
| TSRL | 0.9 | 0.085 | |
| NSR | -0.925 | 0.071 | |
| PSRL | 0.929 | 0.021 | |
| RSGA | 0.913 | 0.053 | |
| Pot Grain | 0.077 | 0.555 | |

FIGURE S1 Relationship between coefficient of variation (CV) of grain yield in soil-filled chamber experiment and field yield of 8 cultivars of spring wheat. * p ≤ 0.05
